# Author’s Response to Peer Reviews of “Real-Time Health Monitoring Using 5G Networks: Deep Learning–Based Architecture for Remote Patient Care”

**DOI:** 10.2196/83473

**Published:** 2025-10-01

**Authors:** Iqra Batool

**Affiliations:** 1 Department of Computer Science, Western University, 1151 Richmond St, London, ON, N6A 3K, Canada, 1 5196613550

**Keywords:** 5G, real-time patient monitoring, vital signs, prediction, deep learning, machine learning

*This is the authors’ response to peer-review reports for “Real-Time Health Monitoring Using 5G Networks: Deep Learning–Based Architecture for Remote Patient Care.”*

## Round 1 Review

### Reviewer S [1]

We thank Reviewer S for their constructive feedback and positive evaluation of our work [2]. Below, we provide a detailed response to each comment and indicate where these concerns have been addressed in our revised manuscript.

*The combination of a convolutional neural network/long short-term memory model with 5G ultra-reliable low latency communication enables real-time monitoring with high accuracy and low latency. Achieving 96.5% accuracy for vital sign prediction demonstrates the effectiveness of the proposed model.*

**Response:** We appreciate the reviewer’s recognition of our system’s performance. The 96.5% accuracy is detailed in section 5 (Results and Analysis), with comprehensive performance metrics shown in Table 1, demonstrating mean absolute error values of 1.82%, 2.14%, and 1.95% for heart rate, blood pressure, and respiratory rate, respectively.

*While tested on 1000 patients, analysis of its scalability to larger populations with diverse demographics would improve generalizability.*

**Response:** We have extensively addressed scalability concerns in section 5.5 (System Scalability and Performance Analysis). Our analysis includes the following:

- Computational scalability; linear scaling up to 2000 concurrent patients with graceful degradation beyond this threshold
- Network scalability; support for up to 1000 high-priority patients (intensive care/critical care) and 4000 standard-priority patients simultaneously
- Mathematical modeling; scalability relationship modeled using equation 26
- Diverse patient populations; performance validated across critical care (96.5%), postoperative (95.8%), and general ward patients (97.2%)

*The use of attention mechanisms in the long short-term memory component improves the system’s ability to model dependencies in continuous vital sign monitoring.*

**Response:** The attention mechanism implementation is detailed in section 3.2 (Deep Learning Framework) with mathematical formulations in equations 10 and 11. The 4-head attention mechanism was optimized through Bayesian optimization, as described in section 4.3 (Model Development).

*A more detailed comparison with state-of-the-art remote monitoring systems, including their architectures and limitations, would strengthen the claims.*

**Response:** We have provided comprehensive comparisons in multiple sections:

- Section 4.2—detailed baseline comparison with 3 established systems (ConventionalCare RPM Platform, EdgeMed Smart Monitoring, and NextGen 5G Health Platform)
- Section 5.7—comparative analysis with performance metrics in Table 4
- Statistical validation—statistical significance testing in Table 5, with paired *t*-tests confirming improvements (*P*<.001)
- Architecture details—each baseline system includes mathematical formulations and operational specifications

*Since patient data is transmitted over 5G networks, an evaluation of encryption techniques, data integrity measures, and compliance with health care regulations (eg, the Health Insurance Portability and Accountability Act and the General Data Protection Regulation) should be included.*

**Response:** Security and privacy are comprehensively addressed in section 4.4 (Security Architecture and Data Protection):

- Encryption—AES-256 encryption with mathematical formulation (equation 24)
- Privacy-preserving techniques–differential privacy implementation (equation 25)
- Regulatory compliance—Health Insurance Portability and Accountability Act and General Data Protection Regulation compliance with specific implementation details
- Network security—5G network slicing with isolated communication channels
- Data protection—end-to-end encryption, public key infrastructure management, and role-based access control

*Investigating performance under network congestion, packet loss, or fluctuations in 5G coverage would ensure system reliability.*

**Response:** Network robustness is thoroughly evaluated in section 5.4 (Network Robustness and Reliability Assessment):

- Network congestion—performance maintained at 96.1% accuracy under 50% capacity, 95.3% at 75% congestion
- Packet loss tolerance—system maintains 96.2% accuracy with 1% packet loss, 94.8% with 5% loss
- Coverage fluctuation—automatic fallback mechanisms to 4G with monitoring continuity maintained
- Reliability modeling—mathematical formulation in equation 21

### Reviewer BM [3]

We thank reviewer BM for their careful review and detailed feedback. All identified issues have been addressed in our revised manuscript. Below is our point-by-point response indicating where each concern has been resolved.

#### Major Comments

*Table 3 is not referenced nor commented on in the text. You should add a paragraph explaining the table or delete it.*

**Response:** Table 3 is now properly referenced and explained in section 5.3 (System Latency Analysis). A detailed paragraph has been added explaining the latency breakdown across different processing stages, highlighting that the total pipeline latency of 14.4 ms meets real-time clinical monitoring requirements. Location: section 5.3, paragraph discussing end-to-end latency measurements.

*Table 5 compares the system performance with 3 other systems, A, B, and C, but those systems are never described. They must be commented on in order to compare results.*

**Response:** The 3 baseline systems are comprehensively described in section 4.2 (Baseline Comparison Systems). Additionally, clear cross-references have been added in section 5.7 (Comparative Analysis) directing readers to these detailed descriptions before presenting the comparison results. Locations: detailed descriptions in section 4.2; cross-references added in section 5.7, before Table 4.

#### Minor Comments

*Equation 1 has no label (1) and it is defined twice.*

**Response:** All equations have been properly numbered sequentially throughout the manuscript. Equation 1 now appears only once, with proper labeling, and all subsequent equations follow the correct numerical sequence. Location: throughout the manuscript, starting with equation 1 in section 3.2.

*Figure 4 should be placed after it is called out.*

**Response:** Figure 4 has been repositioned to appear immediately after its first call-out, following standard manuscript formatting guidelines. Location: section 5.1, after the first mention of the performance timeline.

*On page 6, there is a sentence in square brackets.*

**Response:** All editorial comments and square bracket notations have been removed from the manuscript. The document has been thoroughly reviewed to ensure no editorial marks remain. Location: page 6 content has been cleaned and integrated into the proper text.

*Correct the sentence “Figure 4illustrates...” The number 4 and the word “illustrates” are too close.*

**Response:** The spacing error has been corrected to read “Figure 4 illustrates...” with proper spacing between the figure number and text. All similar formatting issues throughout the document have been resolved. Location: section 5.1, Performance Evaluation subsection.

*Table 5 is called out before Table 4. Consequently, they should be switched.*

**Response:** Tables have been reordered to match their sequence in the text. All table numbers and corresponding mentions have been updated accordingly to maintain proper numerical order. Location: tables now appear in correct sequence in section 5.

*The sentence “Table V System Comparison...” seems to be a figure description instead of part of the text. It makes no sense in the place it is located.*

**Response:** All table captions have been properly formatted and positioned according to journal guidelines. Table descriptions have been moved from body text to appropriate caption format. Location: all tables in section 5 now have properly formatted captions.

*The text “(P ! .001)” I presume should be “(P<.001)”*

**Response:** All instances of incorrect mathematical notation such as “p ! 0.001” have been corrected to “*P*<.001”. The entire manuscript has been reviewed for mathematical symbol accuracy. Location: section 5.7, Table 5, and associated statistical analysis text.

## Round 2 Review

### Reviewer BM [3]

First of all, we would like to thank the reviewers for their valuable reviews. We have addressed all the comments, and our detailed responses are below.

*There are some equations with no defined parameters. In equation 16, what are Pij and xij? In equation 17, what is N? In equation 18, what are Bi, Cj, and M? In equation 19, what is Lu? They must be defined.*

**Response:** We acknowledge this critical oversight and have thoroughly revised all equations to include complete parameter definitions. The corrections are as follows, under the Resource Allocation heading:

“The resource allocation for the healthcare slice is optimized using:

*min∑*_*i*_*∑*_*j*_*P*_*ij*_*x*_*ij*_ (16)

subject to:

*∑*_*j*_*x*_*ij*_*=1,∀i∈N* (17)

Where:

*P*_*ij*_=power consumption (Watts) when patient *i* is assigned to server *j*

*x*_*ij*_=binary resource allocation variable (1 if patient *i* is assigned to server *j*, *0* otherwise)

*N*=set of all patients requiring monitoring, N={1,2,...,n}

*M*=set of available edge computing servers, M={1,2,...,m}

*B*_*i*_=bandwidth requirement of patient *i* (Mbps)

*C*_*j*_=computational capacity of server *j* (operations per second)

The resource allocation optimization considers four critical system parameters. Power consumption *P*_*ij*_ affects overall energy efficiency and operational costs of the monitoring infrastructure. The binary allocation variable *x*_*ij*_ governs the distribution of computational resources across the network, ensuring each patient is assigned to exactly one processing server. Bandwidth requirements *B*_*i*_ determine the communication overhead for transmitting vital sign data from each patient, while capacity constraints *C*_*j*_ ensure the system operates within the feasible computational limits of each edge server.

Constraint (17) ensures that each patient is assigned to exactly one server, preventing resource conflicts and ensuring complete coverage. Constraint (18) guarantees that the total computational load assigned to any server does not exceed its processing capacity, maintaining system stability and response time requirements.”

*How are weights wu, wr, and wl calculated or estimated? What are their chosen values? The final performance could change depending on the selection of these parameters, as you are giving more importance to one parameter or another.*

**Response:** This is an important methodological question that we have addressed by adding a dedicated subsection, “Weight Parameter Determination,” under the Resource Allocation section:

*“P(i)=w*_*u*_*U*_*i*_*+w*_*r*_*R*_*i*_*+w*_*l*_*L*_*i*_ (20)

where:

*U*_*i*_ is the urgency factor

*R*_*i*_ is the reliability requirement

*L*_*i*_ is the latency requirement

*w*_*u*_*,w*_*r*_*,w*_*l*_ are corresponding weights

Real-time latency monitoring and dynamic route optimization further enhance the system’s reliability and performance through continuous assessment shown in equation (21):

*R(t)=(1–P*_*e*_*)(1–P*_*l*_*)(1–P*_*u*_*)* (21)

where:

*P*_*e*_ is packet error probability

*P*_*l*_ is packet loss probability

*P*_*u*_ is system unavailability probability

The packet scheduling priority weights in equation (20) were determined through simulation-based optimization using the MIMIC-III clinical database. The optimization objective was to minimize false alarms while maximizing critical event detection accuracy across diverse patient scenarios, formulated as a constrained optimization problem with *w*_*u*_*+ w*_*r*_*+w*_*l*_*=1*

The final optimized weights are:

*w*_*u*_u=0.45 (urgency priority)

*w*_*r*_*r*=0.35 (reliability requirement)

*w*_*l*_=0.20 (latency sensitivity)

Sensitivity analysis confirmed robust performance with less than 2% accuracy degradation under ±10% weight variations. For different clinical contexts, weights are adjusted: ICU patients use *w*_*u*_=0.60 for maximum urgency response, while home monitoring emphasizes reliability with *w*_*r*_=0.50.”

### Minor Comments

*Most of the references are “touching” the previous text. Add a space between text and references. For instance: “...clinical settings[1],[2].” should be “... clinical settings [1], [2].”*

**Response:** We have corrected all reference formatting issues throughout the manuscript. All references now include proper spacing between text and citation numbers. Examples of corrections made:

“clinical settings [1], [2]” ✓ (was “clinical settings [1],[2]“)

“remote healthcare solutions [2], [3]” ✓ (was “solutions [2],[3]”)

“existing communication networks [4], [5]” ✓ (was “networks [4],[5]”)

“particularly poor connectivity [6], [7]” ✓ (was “connectivity [6],[7]”)

This formatting has been standardized throughout the entire manuscript for consistency.

*Figure 2 should be closer to where it is referred to on the previous page.*

**Response:** We have repositioned Figure 2 to appear immediately after its first reference in the text. The figure now appears directly following the paragraph that introduces the system architecture components, improving readability and flow.

## References

[1] Bharadwaj S (2025). Peer review of “Real-Time Health Monitoring Using 5G Networks: Deep Learning–Based Architecture for Remote Patient Care”. JMIRx Med.

[2] Batool I (2025). Real-time health monitoring using 5G networks: deep learning–based architecture for remote patient care. JMIRx Med.

[3] Gonzalez-Canete FJ (2025). Peer review of “Real-Time Health Monitoring Using 5G Networks: Deep Learning–Based Architecture for Remote Patient Care”. JMIRx Med.

